# Targeting Base Excision Repair in Cancer: NQO1-Bioactivatable Drugs Improve Tumor Selectivity and Reduce Treatment Toxicity Through Radiosensitization of Human Cancer

**DOI:** 10.3389/fonc.2020.01575

**Published:** 2020-08-19

**Authors:** Colton L. Starcher, S. Louise Pay, Naveen Singh, I-Ju Yeh, Snehal B. Bhandare, Xiaolin Su, Xiumei Huang, Erik A. Bey, Edward A. Motea, David A. Boothman

**Affiliations:** ^1^Department of Biochemistry and Molecular Biology, IU Simon Cancer Center, Indiana University School of Medicine, Indianapolis, IN, United States; ^2^Department of Radiation Oncology, IU Simon Cancer Center, Indiana University School of Medicine, Indianapolis, IN, United States

**Keywords:** NQO1, PARP1 hyperactivation, ionizing radiation, base excision repair, double-strand break repair, synergy, β-lapachone, abasic sites

## Abstract

Ionizing radiation (IR) creates lethal DNA damage that can effectively kill tumor cells. However, the high dose required for a therapeutic outcome also damages healthy tissue. Thus, a therapeutic strategy with predictive biomarkers to enhance the beneficial effects of IR allowing a dose reduction without losing efficacy is highly desirable. NAD(P)H:quinone oxidoreductase 1 (NQO1) is overexpressed in the majority of recalcitrant solid tumors in comparison with normal tissue. Studies have shown that NQO1 can bioactivate certain quinone molecules (e.g., ortho-naphthoquinone and β-lapachone) to induce a futile redox cycle leading to the formation of oxidative DNA damage, hyperactivation of poly(ADP-ribose) polymerase 1 (PARP1), and catastrophic depletion of NAD^+^ and ATP, which culminates in cellular lethality via NAD^+^-Keresis. However, NQO1-bioactivatable drugs induce methemoglobinemia and hemolytic anemia at high doses. To circumvent this, NQO1-bioactivatable agents have been shown to synergize with PARP1 inhibitors, pyrimidine radiosensitizers, and IR. This therapeutic strategy allows for a reduction in the dose of the combined agents to decrease unwanted side effects by increasing tumor selectivity. In this review, we discuss the mechanisms of radiosensitization between NQO1-bioactivatable drugs and IR with a focus on the involvement of base excision repair (BER). This combination therapeutic strategy presents a unique tumor-selective and minimally toxic approach for targeting solid tumors that overexpress NQO1.

## Introduction

Ionizing radiation induces high levels of single-strand DNA breaks (SSBs), double-strand DNA breaks (DSBs), and oxidized bases via ROS production and DNA–protein cross-links that activate almost all DNA repair pathways (1, 2). Although effective, the toxicity of IR to healthy tissue at a therapeutic dose presents a significant limitation in the clinic (3–5). IR activates the BER pathway, in which DNA glycosylases (e.g., OGG1) create abasic sites and SSBs for base excision and replacement (6). If these SSBs persist, are replicated through, or are within three base pairs of each other, they are converted to DSBs. The presence of one unrepaired DSB has been reported to be lethal (7, 8). Thus, combining IR with an agent that also promotes a significant increase in DNA damage through modified bases and deleterious DSBs preferentially in tumors may effectively reduce the necessary dose of IR in a clinical setting to lessen toxicity to healthy tissues and improve patient outcomes. The use of a tumor-selective drug for this purpose is an attractive possibility.

NAD(P)H:quinone oxidoreductase 1 (NQO1, also called DT-diaphorase) is a phase II two-electron redox enzyme that is highly overexpressed in most solid tumor types compared with most healthy tissues, as shown through studies by Siegel and Ross (9, 10). Ortho-napthoquinones are a unique class of quinone molecules that, unlike other quinones that are conjugated to glutathione and excreted from the cell, are bioactivated specifically by NQO1 to undergo a two-step back-reaction with oxygen (11). In this futile cycle, NQO1 continuously metabolizes the drugs and then reverts them to the parent compound (12). This process causes rapid accumulation of ROS such as superoxide radical and hydrogen peroxide (H_2_O_2_) that permeate the cell and nuclear membrane to cause significant numbers of oxidized bases and SSBs, which consequently lead to the formation of lethal DSBs. Poly(ADP-ribose) polymerase-1 (PARP1) is hyperactivated by this DNA damage, which rapidly depletes NAD^+^ and ATP, causing metabolic catastrophe and cell death via programmed necrosis (termed NAD^+^-Keresis) (13).

Base excision repair is the main repair pathway involved in activating PARP1 during the repair of SSBs and oxidized bases (14). Depleting BER enzymes, such as XRCC1, and modification of apurinic/apyridinic (AP) sites with methoxyamine (MeOX) synergizes with NQO1-bioactivatable drugs, promoting increased DSBs and rapid cell death (15). NQO1-bioactivatable drugs have long been known to synergize with halogenated pyrimidine radiosensitizers (16). More recently, synergy between PARP inhibitors (17) and IR (18, 19) has been shown. The use of NQO1-bioactivatable drugs, therefore, may be a clinically viable approach to reduce the toxicity of IR associated with high doses and also to improve the tumor selectivity of treatment. In this review, we discuss the mechanisms of radiosensitization between low doses of NQO1-bioactivatable drugs and IR—with a focus on the BER repair pathway and PARP1 hyperactivation—and present a case for combination treatment with NQO1-bioactivatable drugs and IR in the clinic.

## NQO1-Bioactivatable Drugs Induce a Specific Form of Programmed Necrosis (NAD^+^-Keresis)

**FIGURE 1:**
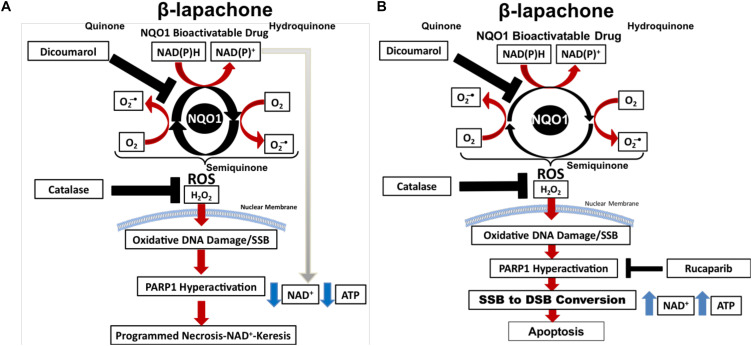
PARP inhibitors switch cell death to apoptosis from programmed necrosis. **(A)** NOQ1 bioactivatable drug β-lap mediates a futile redox cycle with NQO1 detoxifying enzyme, creating a large pool of NAD^+^ and hydrogen peroxide. Hydrogen peroxide formation leads to the formation of oxidized bases and SSBs that induces PARP1 hyperactivation. PARP1 utilizes NAD^+^ for activity, which depletes NAD^+^ and ATP, resulting in NAD^+^-Keresis. **(B)** Addition of PARP inhibitor prevents PARP1 hyperactivation and spares ATP and NAD^+^. Cellular processes can then recycle NAD^+^ back to NAD(P)H, which power more turns of the futile cycle creating even more oxidized bases and SSBs. PARP1 inhibition results in SSB-to-DSB conversion and death by apoptosis.

β-Lapachone (β-lap/ARQ761 in clinical form) is an NQO1-bioactivatable drug derived from lapachone (20), with known antimicrobial (21) and anticancer activity as a single agent (22). The futile redox cycling of β-lap by NQO1 (11) produces ROS-induced DNA damage (Figure 1A), which ultimately leads to cell death via metabolic and bioenergetic catastrophe caused by NAD^+^ and ATP depletion following PARP1 hyperactivation (23). Boothman and colleagues have shown that within 5 min of β-lap treatment, there is a significant calcium flux from the ER to the cytosol (24). Calcium flux from the ER is necessary to activate calpain protease (24) and hyperactivate PARP1; however, the mechanistic role of calcium in PARP1 hyperactivation has yet to be firmly established (25). Within 30 min, the NAD^+^ molecules that are produced during the futile redox cycling of β-lap by NQO1 are rapidly exhausted by hyperactivated PARP1 during the repair of ROS-induced DNA damage and SSBs (17). Depletion of NAD^+^ consequently depletes ATP and induces a specific type of programmed necrosis, termed NAD^+^-Keresis (26). Expression of catalase can spare cellular lethality by neutralizing the effects of hydrogen peroxide (H_2_O_2_) produced by β-lap (an NQO1-bioactivatable agent), confirming the role of ROS formation in toxicity (27). Inhibition of NQO1 activity with a small-molecule inhibitor (e.g., Dicoumarol) or genetically knocking out NQO1 eliminates β-lap lethality, showing the selectivity of β-lap-induced cell death to NQO1-expressing cells (17, 23). Calcium release from the ER can be blocked with the calcium chelator, BAPTA-AM, which prevents PARP1 hyperactivation and spares cancer cells from lethality, further highlighting the role of PARP1 in β-lap-induced cell death (25). When NAD^+^ production is inhibited genetically by depleting NAMPT or pharmacologically with NAMPT inhibitors (e.g., FK866) (26) prior to β-lap treatment in NQO1-overexpressing cancer cells, a synergistic cell death due to compromised NAD^+^ production following PARP hyperactivation highlights the critical role of catastrophic NAD^+^ depletion in NAD^+^-Keresis (Figure 1A) (26).

## Trapping PARP1 on DNA Synergizes With NQO1-Bioactivatable Drugs

There are 17 known PARP proteins (28) that share a common catalytic domain but exhibit differential roles in DNA repair, chromatin structure and modification, transcription, and cell death. PARP proteins catalyze the transfer of one or more ADP-ribose units to substrate proteins through a process known as mono- or poly(ADP) ribosylation, respectively (29). Of particular importance to the NQO1-bioactivatable drug field is PARP1, which is required for both BER and NER to recruit and activate SSB repair proteins (30). In BER, PARP1 forms a critical complex with DNA ligase III, XRCC1, and DNA pol β (30).

In BRCA1/2-deficient breast and ovarian cancers—which are deficient in homologous recombination (HR) to repair DSBs—PARP1 inhibitors are an effective therapeutic strategy targeting repair of SSBs and BER (31). PARP trapping agents (e.g., talazoparib, rucaparib, and olaparib) are the most effective PARP1-targeting drugs, which trap PARP1/2 on the DNA by binding at the active site, preventing its interaction with NAD^+^ and therefore preventing dissociation via the auto-PARylation domain (32). PARP trapping prevents the recruitment of proteins needed to complete BER, leaving unrepaired SSBs that are then converted to lethal DSBs upon collision with the replication and transcription machineries (31, 32).

Recently, we reported that PARP-trapping agents Rucaparib and Talazoparib synergize with β-lap in NQO1+ lung, pancreatic, and TNBC cell lines and *in vivo* models of NSCLC (17). Sublethal β-lap doses showed significant synergy with non-toxic doses of PARP inhibitor Rucaparib in multiple cancer types, and up to 60 different NSCLC cell lines (17). Synergy occurred regardless of oncogenic and tumor-repressor mutations and was entirely NQO1-dependent in all cell types (17), according to the gold standard combinatorial index obtained using the Chou and Talalay method (Figure 1B) (33).

Mechanistically, the addition of non-toxic doses of PARP inhibitor (e.g., Rucaparib) to sublethal β-lap doses prevents the loss of NAD^+^ and ATP (17). No PARylation of PARP1 occurred in this instance; however, DSBs significantly increased, indicating a β-lap-mediated SSB-to-DSB conversion (17). NAD^+^ and ATP sparing allows for more oxygen consumption during the futile redox cycling of NQO1-bioactivatable agents, increasing the formation of oxidized bases and unrepaired SSBs (17). This process overwhelms the DNA damage response and repair (17). ATP is then used to initiate caspase-dependent apoptosis, which is in contrast with the NAD^+^-Keresis observed with β-lap monotherapy (17). PARP inhibitors, therefore, enhance DNA damage caused by NQO1-bioactivatable drugs and switch cell death from programmed necrosis to apoptosis (17). This is significant as necrosis may cause inflammation and lead to complications, whereas apoptosis does not. Combining β-lap with PARP1 inhibitors, therefore, reduces the toxicity of the drug in addition to enhancing its mechanism of action, making it more attractive for clinical application.

## BER Is the Major DNA Repair Pathway Involved in the NQO1-Bioactivatable Drug Mechanism of Action

Base excision repair resolves non-distorting DNA lesions resulting from alkylation, oxidation, depurine/pyrimidination, and deamination, which can be drug-induced or occur from exposure to environmental toxins. There are two types of BER: short patch that repairs a single damaged base and long patch that repairs up to three damaged bases (34). The typical mammalian BER pathway occurs as follows: DNA glycosylases detect damaged bases and cleave the glycosidic bond holding the damaged base to the DNA backbone, creating an apurinic/apyridinic site (AP site). AP sites are cleaved by AP endonucleases (APE1/APE2), allowing DNA pol β to fill the site with the appropriate base (35, 36). Mechanistically, APE1 provides a significant portion of the endonuclease activity, while APE2 provides some endonuclease activity and a large portion of exonuclease activity (34). Both APE1 and APE2 provide proofreading capabilities for pol β to reduce error rates (37). DNA ligase then seals up this stretch of DNA to finalize the DNA repair (35).

Hydrogen peroxide induced by β-lap permeates the nucleus and oxidizes nucleotides, particularly guanine bases (e.g., 8-oxo-guanine or 8-oxoG) (15). Oxidized guanine (8-oxoG) formed during treatment with β-lap recruits DNA glycosylase OGG1, which, combined with APE1/2, produces a SSB that activates PARP1 during BER (15). OGG1 recognizes the oxidized lesion, cleaves at the 3′ end, and removes the lesion, in a reaction that is catalyzed by ATP (38). It has been shown that silencing OGG1 prevents 8-oxoG recognition and increases the overall amount of 8-oxoG incorporated into DNA (32). This prevents PARP1 hyperactivation, thus abrogating NAD^+^/ATP loss and β-lap-mediated lethality (15). This is an important finding and a potential route of resistance in the clinic to NQO1-bioactivatable drugs.

Silencing the key BER protein, XRCC1, synergizes with NQO1-bioactivatable drugs in PDAC cell lines, further indicating that BER inactivation plays a critical role in β-lap toxicity (15). XRCC1 is a scaffolding protein required for clearing oxidized bases (39). PARylated-PARP1 bound to SSBs recruits XRCC1 through the BRCT 1 domain and forms a complex consisting of XRCC1 (40), DNA pol β (41), DNA Ligase IIIα (42), and APE1 (43). Without XRCC1, DNA base lesions and SSBs cannot be repaired. In addition to mediating synthetic lethality, XRCC1 knockdown also depletes NAD^+^ levels at a higher rate than with β-lap alone (15). XRCC1 knockdown is currently known to be embryonic lethal and essential for mouse development (44). NQO1-bioactivatable drug synergy with BER deficiencies in PDAC cells indicates a potential for these drugs to be beneficial in targeting pancreatic cancer.

Silencing OGG1 spared β-lap-mediated lethality in PDAC, compared with XRCC1 knockdown (15). It is thought that knockdown of OGG1 glycosylase protects PDAC cells from death because BER is not activated if the scanning glycosylase is non-functional. This suggests an important role for different proteins in BER with regard to solid tumors. Alteration of expression or mutation of different proteins in the BER pathway can either sensitize or protect cancer cells from NQO1-bioactivatable drug-mediated lethality.

**FIGURE 2:**
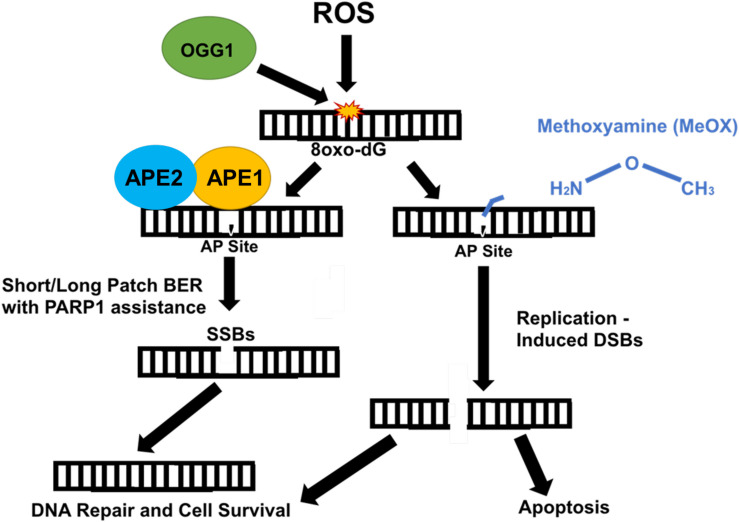
Methoxyamine permanently modifies AP sites and prevents their repair by BER. Reactive oxygen species (ROS) modify guanine bases in DNA, which are recognized and cut by type II DNA glycosylase, OGG1, creating an AP site. Due to OGG1 activity as a type II glycosylase, OGG1 is capable of cleaving the AP site directly. However, under normal circumstances, APE1/APE2 come in and cut, cleaving the AP site, which is repaired either through short patch or long patch BER via PARP1 and XRCC1 scaffold protein that recruit appropriate proteins (i.e., Pol β) necessary for repair. APE1/APE2 both provide proofreading for pol-β to prevent errors in repair (37). APE1 provides most of the endonuclease activity compared with APE2, which provides some endonuclease activity and a large amount of exonuclease activity (36). Methoxyamine permanently modifies the AP site preventing PARP1 and other necessary proteins from accessing and fixing SSBs. These SSBs are converted to DSBs and result in cell death.

Methoxyamine is an AP site modifier (Figure 2) used to sensitize temozolomide-resistant glioblastoma (45) and ovarian cancer (46). MeOX modification of AP sites prevents their degradation, thus mitigating sodium hydroxide-mediated hydrolysis of the DNA backbone, preventing AP site cleavage, blocking AP endonuclease action, and preventing BER, resulting in cell death (47). MeOX synergizes with β-lap, increasing the number and persistence of AP sites, PARylation, and DSBs in PDAC. This is specific to β-lap, as co-treatment with NQO1-inhibitor dicoumarol abrogates AP site formation. β-lap and MeOX were also shown to synergize and ultimately reduce tumor volume in 33% of PDAC murine xenografts (15).

## Double-Strand Break and BER Play a Critical Role in Tumor Response to IR

Ionizing radiation is one of the most common and effective methods for treating solid tumors in cancer patients. IR damages DNA directly by causing ionization in DNA itself or indirectly by ionizing the surrounding water resulting in aqueous free radicals that can react with DNA. Inducing significant DNA damage by IR over several treatments results in cancer cell death; however, there are significant drawbacks to this approach, including limitations on the number of IR dose a person can receive in a lifetime, costs, the need for special diets, and serious side effects arising from healthy tissue damage (48). IR produces 1000 SSBs, 40 DSBs, 700 altered thymine bases, 700 8-oxoG base alterations, and 150 DNA–protein cross-links per gray (Gy) (49, 50). The resistance of cancer cells is considered to be determined by the efficacy of DSB repair (51, 52).

**FIGURE 3:**
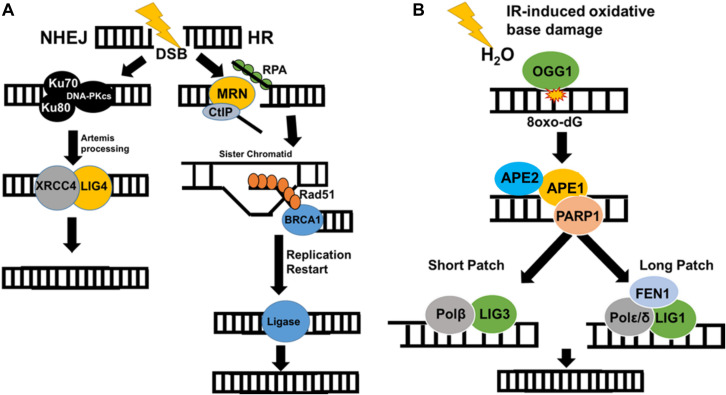
Ionizing radiation induces a wide variety of DNA damage. **(A)** IR causes dsDNA breaks that are repaired by HR (in S/G2 phase) or NHEJ (all phases of cell cycle). In HR, the RPA complex and BRC proteins form a scaffold complex with the sister chromatid and use it as a template to correct damage without error. NHEJ utilizes the KU70/80/DNA-PKc complex to quickly combine and ligate double-strand breaks. **(B)** IR-mediated radiolysis of water leads to ROS formation, which then creates base damage through oxidation. BER then repairs these lesions through the use of a type II DNA glycosylase (OGG1), AP endonuclease (APE1/APE2), PARP1, DNA polymerase, and ligase. This occurs through either short patch or long patch BER.

Ionizing radiation-induced DSBs activate DSB repair via NHEJ and HR (Figure 3A). NHEJ occurs in all phases of the cell cycle (53), which is a quick and easy way to fix massive levels of dsDNA breaks, and is utilized for V(D)J recombination for the human immune system (52). During this process, Ku70 and Ku80 heterodimers bind the end of the double-stranded DNA breaks and form a complex to protect and recruit DNA-PKcs (54) to the site of the damage. XRCC4 binds to the Ku dimers through Ku70 mediating the attachment of other proteins necessary to fix the damage (e.g., PNKP, APLF, and XLF) (55). Artemis trims the complex ends (56) of the DSBs for efficient ligation of DNA ends by DNA Ligase IV/XRCC4 complex (Figure 3A) (57) to complete the repair of DSB.

Homologous recombination occurs specifically in the S/G2 phase of the cell cycle and uses the sister chromatid as a template to complete repair of DSBs. HR is known as an error-free repair and minimizes the chances of mutations of functional genes (58). After IR creates a DSB, the MRN complex (Mre11-Rad50-Nbs1) will bind to the ends of the breaks and recruits the CtIP complex exonuclease to create free ends that can be modified (59). Single-stranded DNA-binding protein (hSSB1) and RPA bind the free single-stranded DNA after resection to prevent the degradation, improper hybridization, or combination of DNA ends (60). These proteins then bind to BRCA scaffold proteins (44) that load Rad51 proteins, which are responsible for creating a Holliday Junction to align homologous sequences with the sister chromatid strand (Figure 3A) (61). Rad51 is then released from the RPA complex (62); DNA is synthesized and then ligated by DNA Ligase I (63). Up-regulation of HR or NHEJ can lead to IR resistance and neoplastic growth.

A significant portion of DNA lesions created by IR is through a water-mediated radiolysis reaction (64). Radiolysis of water causes significant ROS production including extremely reactive hydroxyl (•OH) radicals close to DNA resulting in damage that is primarily repaired by BER (Figure 3B) (65). BER may result in DSBs being formed by replication through lesions. If multiple oxidized lesions are within 3 base pairs of each other and BER enzymes cut these lesions out, this will result in the formation of DSBs (65). In addition, mutations in bacterial BER proteins have been known to confer resistance to IR up to 250 Gy, suggesting that BER is necessary for sensitization of IR (65).

## Sublethal Doses of β-Lap Radiosensitize NSCLC Cells to Low-Dose Radiation Therapy

**FIGURE 4:**
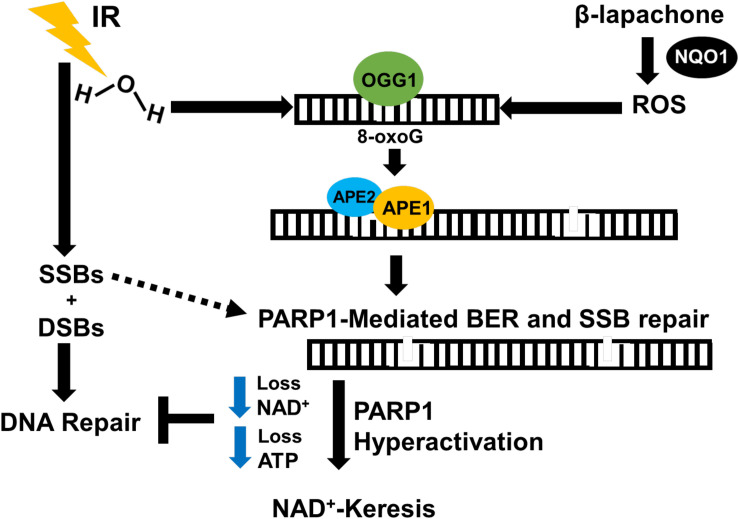
Proposed mechanism of IR and β-lap radiosensitization. NSCLC tumors contain high levels of NQO1 compared to normal tissues. In the presence of NQO1, β-lap causes ROS-induced oxidative base DNA damage, which eventually leads to the formation of SSBs that activate PARP1. IR induces massive SSBs through contact with DNA and oxidized bases due to water radiolysis that require PARP1 and BER to resolve. This combination therapy pushes cumulative amount of DNA damage high enough that overwhelms and hyperactivates PARP1 during DNA damage response and repair, leading to programmed necrosis. Thus, NQO1 may be used as a predictive biomarker for selective targeting of NQO1-overexpressing cancers with low-dose IR in combination with NQO1-bioactivatable agents as radiosensitizers.

In A549 and H1650 NSCLC cell lines, a sublethal dose of β-lap causes significant sensitization to low-dose radiation therapy, leading to a remarkable increase in cell death. Monotherapy with sublethal β-lap induces minimal DSBs, and low-dose radiation monotherapy induces characteristic increase in DSBs followed by efficient repair (18). Combination therapy with low doses of β-lap and radiation therapy, however, promotes rapid and sustained 53BP1 and gamma-H2AX foci formation that is consistent with DSB formation and compromised DSB repair (18). In NQO1+ NSCLC luciferase murine models, β-lap and IR combination therapy reduces tumor volume and increases survival up to 70% in comparison with either agents alone. Tumor tissues from mice treated with IR and β-lap demonstrated enhanced PAR and gamma-H2AX (pS139-H2AX, surrogate marker for DSBs) level compared with monotherapy, as well as decreased NAD^+^ and ATP levels (18). Normal tissues, which generally overexpress catalase and lack NQO1, were unaffected by co-treatment. The BER pathway may play a crucial role in this sensitization since β-lap treatment engages BER and PARP hyperactivation, and IR also activates BER. Mechanistically, we hypothesize that the combination of IR and β-lap treatment creates cumulative clusters of oxidative DNA damage and SSBs that result in severe PARP1 hyperactivation (inactive form of PARP1), which compromises BER and SSB repair. SSBs that are unrepaired are eventually converted to lethal unrepaired DSBs due to the lack of NAD^+^ and ATP molecules available to activate the efficient repair of SSBs and DSBs (Figure 4). Since most solid tumors overexpress NQO1, combining low-dose radiation with a sublethal concentration of β-lap may enhance tumor-selective and targeted killing and improve patient safety by lowering the overall doses of both agents. Using NQO1 as a predictive biomarker, this combination treatment strategy may reduce the impact of treatment on a patient’s lifetime exposure to IR, may cut the costs associated with cancer treatment, and potentially reduce the amount of time needed for therapeutic response.

## Outlook/Future

Further work is required to fully determine the critical role of BER in IR and β-lap combination therapy. We have previously shown that loss of specific BER factors potentiates the lethality of an NQO1-bioactivatable agent, β-lap, selectively in NQO1-overexpressing solid tumors. In fact, inhibition of PARP1—a critical factor involved in DNA damage response and repair of modified DNA bases and SSBs—prior to treatment with NQO1-bioactivatable drug causes a synergistic cancer cell death. Thus, we hypothesize that PARP1-mediated BER and SSB repair are the main DNA repair pathways that are activated by β-lap, which promotes severe PARP1 hyperactivation and subsequent lethality at high doses. PARP inhibitors in combination with IR *and* NQO1-bioactivatable drugs may further enhance synergy seen previously (6); however, three-drug combinations are currently rare. Overall, combining low-dose radiation therapy with NQO1-bioactivatable drugs may be a viable, less toxic, and more tumor-selective strategy for treatment of various solid tumors that overexpress a predictive biomarker, NQO1.

## Author Contributions

DB, prior to his death, directed the review’s thematic points regarding the synergistic value of IR or PARP inhibitors in combination with NQO1-bioactivatable therapies in the treatment of various NQO1-positive cancers. Following DB’s death, CS (DB’s graduate student), EB, EM, and XH (DB’s previous postdoctoral fellows, now faculty at IU School of Medicine) worked to write, edit, and review the manuscript. CS and EM took the lead in writing and made all the figures. EB, XH, NS, SB, SP, I-JY, and XS contributed to the editing of the review article. All authors contributed to the article and approved the submitted version.

## Conflict of Interest

The authors declare that the research was conducted in the absence of any commercial or financial relationships that could be construed as a potential conflict of interest.

## Abbreviations

53BP1: tumor suppressor p53 binding protein 1
8-oxoG: 8-Oxoguanine
AP site: apurinic/apyrimidinic site
APE1: apurinic/apyrimidinic endonuclease 1
APE2: apurinic/apyrimidinic endonuclease 2
ATP: adenosine triphosphate
BAPTA-AM: (1,2-bis(o-aminophenoxy)ethane-N,N,N’,N’-tetraacetic acid)
BER: base excision repair
CtIP Complex: complex involved with MRN and BRCA as a scaffold
DIC: dicoumarol
DNA pol β: DNA polymerase beta
DNA-PKcs, DNA dependent protein kinase: catalytic subunit
DSB: double-strand break
ER: endoplasmic reticulum
gH2AX: H2A histone family member X (phosphorylated Serine 139)
Gy: gray of ionizing radiation
HAN: head and neck cancer
IR: ionizing radiation
Ku70/Ku80: XRCC5/XRCC6
MeOX: methoxyamine
MRN: complex of Mre11
Rad50: and Nbs1 involved in end processing
NAD^+^: nicotinamide adenine dinucleotide
NAMPT: nicotinamide phosphoribosyltransferase
NER: nucleotide excision repair
NHEJ: non-homologous end-joining
NQO1: NAD(P)H:quinone oxidoreductase 1
NSCLC: non-small cell lung cancer
OGG1: 8-Oxoguanine DNA glycosylase 1
PARylation: poly-ADP-ribosylation
PDAC: pancreatic ductal adenocarcinoma
ROS: reactive oxygen species
RPA: replication protein A
SSB: single-strand break
ssDNA: single-strand DNA
TNBC: triple-negative breast cancer
XRCC1: X-ray repair cross-complementing protein 1
XRCC4: X-ray repair cross-complementing protein 4.
